# Scleredema Associated With IgG/κ Monoclonal Gammopathy of Clinical Significance Successfully Treated With Daratumumab Monotherapy: A Case Report

**DOI:** 10.1002/jha2.70262

**Published:** 2026-03-11

**Authors:** Davide Marcolongo, Alessandra Bressan, Elisabetta Zanatta, Mauro S. A. Alaibac, Ilaria Gianesello, Gianni Binotto, Laura Pavan, Angelo Paolo Dei Tos, Livio Trentin, Fabrizio Vianello

**Affiliations:** ^1^ Hematology Unit Department of Medicine University Hospital of Padua Padua Italy; ^2^ Institute of Pathology Department of Medicine University Hospital of Padua Padua Italy; ^3^ Rheumatology Unit Department of Medicine University Hospital of Padua Padua Italy; ^4^ Dermatology Unit Department of Medicine University Hospital of Padua Padua Italy

**Keywords:** cutaneous MGCS, case report, daratumumab, IgG/κ, monoclonal gammopathy of clinical significance (MGCS), scleredema adultorum

## Abstract

**Background:**

Cutaneous monoclonal gammopathy of clinical significance (MGCS) is rare and may present with scleredema‐like fibrosing skin disease.

**Case:**

A 59‐year‐old man developed progressive induration of the upper body. Laboratory studies revealed an IgG/κ monoclonal protein, and skin biopsy showed dermal thickening with mucin deposition. He was refractory to corticosteroids, immunosuppressants, IVIG, plasmapheresis, and bortezomib.

**Results:**

Daratumumab monotherapy led to rapid improvement after 4–5 cycles, sustained over 20 cycles, with mobility recovery, mRSS reduction, paraprotein disappearance, and partial histologic regression.

**Conclusion:**

Daratumumab may represent an effective clone‐directed therapy for refractory MGCS‐associated scleredema.

**Trial Registration**: The authors have confirmed clinical trial registration is not needed for this submission

## 1 Introduction

Monoclonal gammopathy of clinical significance (MGCS) comprises a group of disorders in which a small plasma‐cell or B‐cell clone produces a monoclonal protein that directly contributes to tissue or organ injury [1, 2]. Unlike monoclonal gammopathy of undetermined significance (MGUS), MGCS is defined by its pathogenicity despite the absence of multiple myeloma or lymphoma. Depending on the organ affected, MGCS can manifest as renal, neurologic, ocular, or dermatologic disease. Cutaneous MGCS is uncommon and includes scleromyxedema, necrobiotic xanthogranuloma, and scleredema adultorum of Buschke [3]. These conditions share a pathogenic contribution of the monoclonal protein and are often characterized by partial or transient responses to conventional immunosuppressive therapy. Scleredema associated with an IgG/κ paraprotein is exceptionally rare, typically severe, and may be highly refractory to treatment. Histologically, scleredema is characterized by diffuse dermal thickening and mucin deposition [4]. We report a case of IgG/κ MGCS‐associated scleredema showing a rapid, durable clinical, hematologic, and histologic response to daratumumab monotherapy. This case is unique because it documents, for the first time, a robust and durable clinical, hematologic, and histologic response to daratumumab monotherapy in scleredema associated with MGCS. To our knowledge, this is the first documented report of sustained disease modification in this phenotype, expanding the spectrum of MGCS‐related cutaneous disorders responsive to anti‐plasma‐cell therapy.

## 2 Case Presentation

**FIGURE 1:**
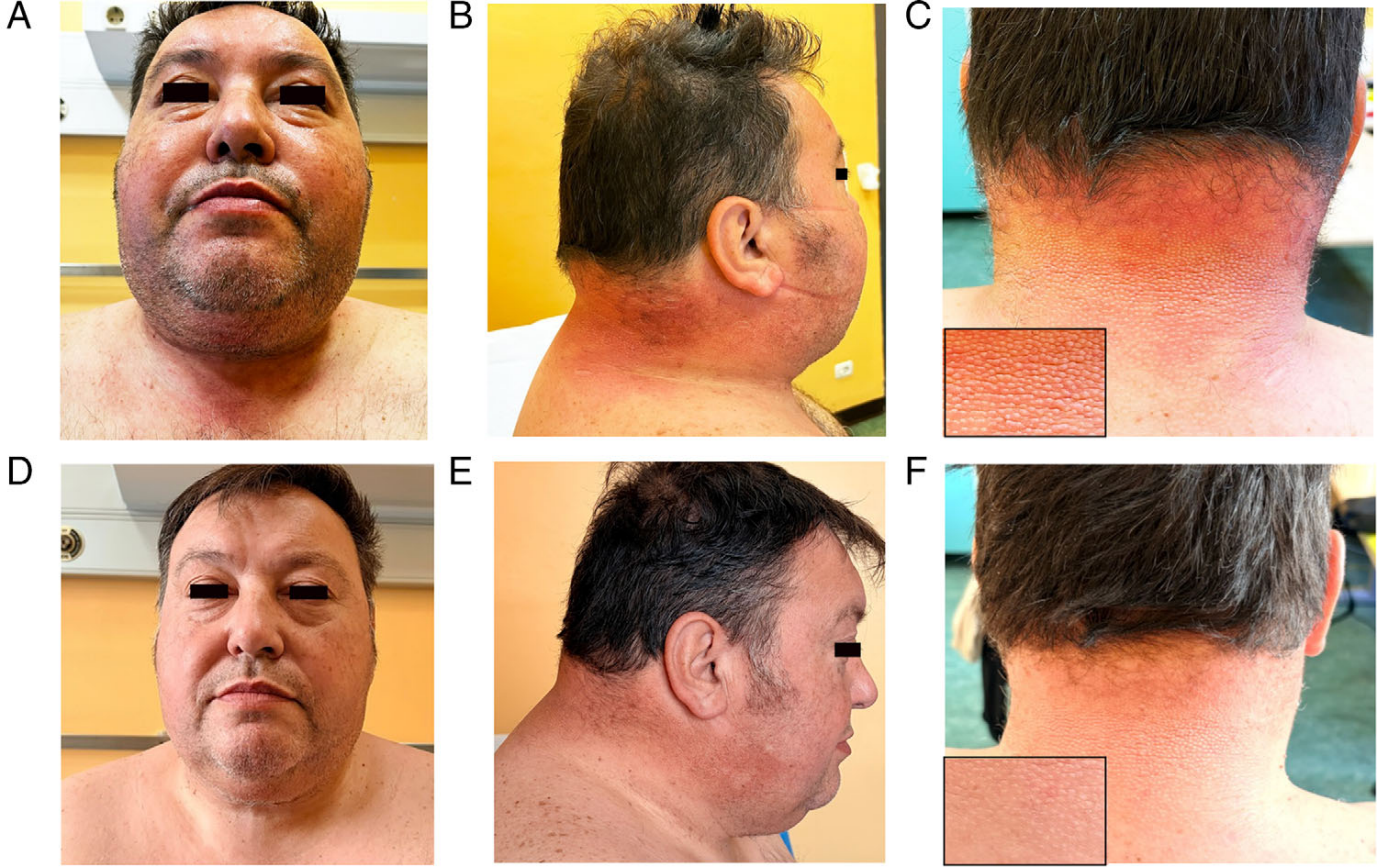
Clinical appearance before and after daratumumab therapy. Upper row (A–C): Baseline. Marked skin thickening and induration of the face, neck, and upper back, with reduced cervical and shoulder mobility. Facial skin appears tense and edematous, with a mask‐like appearance. Lateral and posterior views show pronounced dermal thickening, erythema, and coarse accentuation of the cutaneous texture of the posterior neck, with a clear “peau d'orange” pattern (inset). Lower row (D–F): Month 12 of daratumumab therapy. Substantial clinical improvement with reduction of facial and cervical skin thickening, softer and more mobile skin, and improved definition of the cervical profile. Posterior neck views show a clear decrease in dermal thickening and a marked attenuation, though not complete disappearance, of the previously prominent cutaneous texture. The inset highlights residual but significantly reduced peau d'orange changes.

A 59‐year‐old man presented with a 2‐year history of gradually progressive skin thickening and induration of the upper back, shoulders, and neck. The condition extended to the trunk and face, causing a mask‐like facies and severe limitation of cervical and shoulder motion (Figure 1). He denied Raynaud's phenomenon, digital ulcers, dysphagia, or systemic symptoms. The patient had no significant past medical history aside from Class III obesity and no prior autoimmune, metabolic, or hematologic disorders. He was not taking chronic medications and had no history of diabetes, thyroid dysfunction, or malignancy. There was no family history of plasma‐cell dyscrasias, connective tissue disease, or other heritable disorders. The patient reported functional limitation from skin rigidity but no psychiatric issues or social vulnerabilities. No occupational or environmental exposures relevant to sclerodema‐like disorders were identified.

**FIGURE 2:**
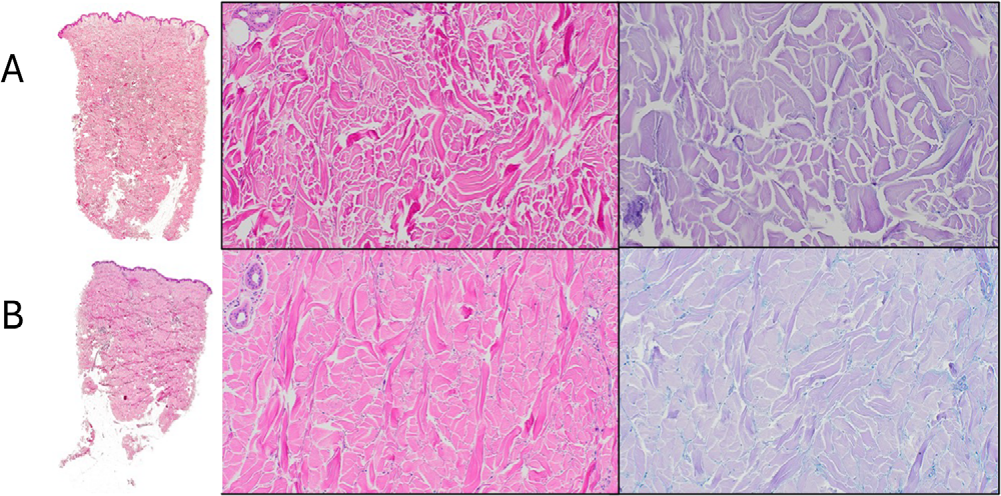
Histological findings: Comparison between the 2022 (A) and 2025 (B) biopsies(H&E×1.25, H&E×10, Alcian PAS×10).In the former, thickening of the reticular dermis and widening of the spaces between collagen bundles are more pronounced.

Laboratory tests showed normal inflammatory markers, creatine kinase, and autoimmune serology (ANA, ENA, Scl‐70, anti‐centromere). Anti‐PM/Scl antibodies were mildly positive. Serum protein electrophoresis identified an IgG/κ monoclonal spike (10.4 g/L) with a κ/λ ratio of 2.1. There was no anemia, renal impairment, hypercalcemia, or lytic bone lesions. Bone marrow biopsy was technically unfeasible; however, cytological smear and flow cytometry of bone marrow blood revealed a small abnormal plasma‐cell population (0.4% of nucleated cells), expressing CD38, CD138, CD56, CD117, and CD45, with partial CD19 expression—supporting the presence of a minor clonal plasma‐cell process consistent with MGCS. Skin biopsy (Figure 2) demonstrated thickening of the reticular dermis, reduction of adnexal structures, and mucin deposition within widened collagen bundles, highlighted by Alcian PAS staining. The modified Rodnan Skin Score (mRSS) [5], a validated semi‐quantitative clinical tool used to assess skin thickness by palpation across 17 body areas (total Score 0–51), was 34. CT imaging showed no visceral involvement.

The patient was refractory to high‐dose corticosteroids, methotrexate, mycophenolate mofetil, intravenous immunoglobulin, while plasmapheresis yielded only temporary benefit. A 2‐month course of weekly bortezomib, administered as single‐agent therapy targeting the small plasma‐cell clone consistent with MGCS rather than overt plasma‐cell malignancy, was ineffective in improving the cutaneous manifestations. Daratumumab monotherapy was initiated (16 mg/kg weekly × 8, biweekly × 4, and then monthly). Intravenous administration was preferred because extensive skin thickening and fibrosis involving potential injection sites raised concerns about the feasibility and consistency of subcutaneous drug delivery.

Adherence was confirmed through infusion attendance logs; tolerability was assessed at each visit via clinical examination, vital signs, infusion‐related reaction monitoring, laboratory testing, and patient‐reported symptoms. Clinical improvement became evident after 4–5 cycles, with reduced stiffness and progressive recovery of cervical and shoulder motion. A short trial of lenalidomide was discontinued due to limb and facial pain. After 20 cycles, the patient exhibited durable benefit, including near‐complete restoration of shoulder mobility and thoracic expansion, with an mRSS improvement from 34 to 25. The IgG/κ monoclonal component became undetectable after three cycles. No infusion‐related reactions occurred. At the time of manuscript preparation, daratumumab therapy was ongoing.

## 3 Discussion

Cutaneous MGCS comprises a heterogeneous group of disorders driven by pathogenic monoclonal immunoglobulins [2]. These conditions may mimic autoimmune or fibrosing dermatoses and are often refractory to standard immunosuppression, contributing to diagnostic challenges and delayed therapy. In this case, the absence of serologic markers and systemic sclerosis features, combined with detection of an IgG/κ monoclonal protein and clonal plasma cells, supported the diagnosis of MGCS‐associated scleredema.

Monoclonal gammopathy‐associated cutaneous disorders are often conceptualized as paraneoplastic phenomena rather than conditions caused by direct tissue infiltration or immunoglobulin deposition. Scleredema and scleromyxedema, in particular, have been classified among dermatoses strongly associated with monoclonal gammopathy despite the absence of demonstrable monoclonal protein deposition in most cases [6]. Their pathogenesis remains incompletely understood but is thought to involve cytokine‐mediated fibroblast activation driven by the underlying plasma‐cell clone, resulting in collagen deposition, mucin accumulation, and progressive dermal thickening. In our patient, inflammatory markers such as C‐reactive protein were within normal limits, and specific cytokine measurements were not performed; therefore, this pathogenetic hypothesis remains speculative in our case and is based on previously reported models rather than direct evidence [7, 8]. Within this framework, the absence of detectable IgG/κ deposits in our patient does not exclude a pathogenic role of the monoclonal gammopathy. Instead, the parallel clinical improvement and disappearance of the monoclonal component following daratumumab therapy support a plausible biologic link consistent with a MGCS‐related dermatosis.

Evidence for daratumumab in cutaneous MGCS remains limited. Successful responses have been reported in scleromyxedema [9, 10]. Only one previous case of MGCS‐associated skin disease has documented rapid and clinically meaningful improvement following daratumumab therapy after failure of conventional treatments [9]. Daratumumab has also demonstrated activity in necrobiotic xanthogranuloma and related granulomatous dermatoses associated with monoclonal gammopathy, with Bertuglia et al. describing partial or complete regression of granulomatous plaques in parallel with suppression of the plasma‐cell clone [11]. Furthermore, daratumumab‐induced elimination of a pathogenic clone has been shown to provide sustained control of MGCS‐related acquired C1‐inhibitor deficiency [12]. These findings align with the comprehensive review by Claveau et al., which highlighted the central role of clone‐directed therapy in treating cutaneous MGCS and emphasized the potential role of anti‐CD38 therapy across diverse MGCS phenotypes [3]. Notably, no published reports describe a scleredema phenotype analogous to the presentation in our patient.

The clinical response to daratumumab likely reflects both effective plasma‐cell clone suppression and broader immunomodulatory effects of anti‐CD38 therapy, which may be relevant in paraneoplastic or immune‐mediated dermatoses. The limited response to prior bortezomib therapy may relate to biological heterogeneity, depth of clonal suppression, or treatment‐related factors; subcutaneous administration in the presence of marked dermal fibrosis may theoretically have influenced drug absorption, although this remains speculative.

To our knowledge, this is the first documented case in which daratumumab produced a sustained clinical response and histologic benefit in scleredema‐associated MGCS. The parallel disappearance of the IgG/κ monoclonal component is consistent with a plausible pathogenetic link between clonal suppression and dermatological improvement. As a single‐patient report without bone marrow histology, generalizability is limited, and the long‐term durability of response in MGCS‐associated scleredema remains unknown. This case highlights the need to consider MGCS in a patient with atypical, antibody‐negative, treatment‐refractory fibrosing dermatoses and supports early implementation of clone‐directed therapy to prevent irreversible structural remodeling and functional decline.

## 4 Patient Perspective

“Before treatment, I could barely lift my arms or take a deep breath. My skin felt rigid and heavy. After a few cycles of daratumumab, the stiffness started to ease. Now, after many months, I feel much better and can move and breathe more comfortably.”

## Author Contributions

All authors contributed to the conception, drafting, and critical revision of the manuscript. All authors approved the final version and agree to be accountable for all aspects of the work.

## Funding

The authors have nothing to report.

## Ethics Statement

This study was conducted in accordance with the principles of the Declaration of Helsinki.

## Consent

Written informed consent for publication of clinical details and images was obtained from the patient.

## Conflicts of Interest

The authors declare no conflicts of interest.

## Data Availability

All data relevant to this case report are included in the article. Additional details are available from the corresponding author on reasonable request.

## References

[1] A. Dispenzieri , “Monoclonal Gammopathies of Clinical Significance,” Hematology 2020, no. 1 (2020): 380–388, 10.1182/hematology.2020000122. 33275738 PMC7727544

[2] J. P. Fermand , F. Bridoux , A. Dispenzieri , et al., “Monoclonal Gammopathy of Clinical Significance: A Novel Concept With Therapeutic Implications,” Blood 132, no. 14 (2018): 1478–1485, 10.1182/blood-2018-04-839480. 30012636

[3] J. S. Claveau , D. A. Wetter , and S. Kumar , “Cutaneous Manifestations of Monoclonal Gammopathy,” Blood Cancer Journal 12, no. 4 (2022): 58, 10.1038/s41408-022-00661-1. 35411042 PMC9001632

[4] F. Rongioletti , F. Kaiser , E. Cinotti , et al., “Scleredema. A Multicentre Study of Characteristics, Comorbidities, Course and Therapy in 44 Patients,” Journal of the European Academy of Dermatology and Venereology 29, no. 12 (2015): 2399–2404, 10.1111/jdv.13272. 26304054

[5] P. Clements , P. Lachenbruch , J. Siebold , et al., “Inter and Intraobserver Variability of Total Skin Thickness Score (Modified Rodnan TSS) in Systemic Sclerosis,” Journal of Rheumatology 22, no. 7 (1995): 1281–1285. 7562759

[6] M. S. Daoud , J. A. Lust , R. A. Kyle , and M. R. Pittelkow , “Monoclonal Gammopathies and Associated Skin Disorders,” Journal of the American Academy of Dermatology 40, no. 4 (1999): 507–535, 10.1016/S0190-9622(99)70434-2. 10188670

[7] L. Quartuccio , V. Manfrè , E. Treppo , et al., “Monoclonal Gammopathy of Rheumatologic Significance (MGRhS): A Systemic Vision of Clonal Disorders With Multiple Organ Involvement,” Autoimmunity Reviews 24, no. 11 (2025): 103895, 10.1016/j.autrev.2025.103895. 40744142

[8] N. Dumoitier , B. Chaigne , A. Régent , et al., “Scleroderma Peripheral B Lymphocytes Secrete Interleukin‐6 and Transforming Growth Factor β and Activate Fibroblasts,” Arthritis & Rheumatology 69, no. 5 (2017): 1078–1089, 10.1002/art.40016. 27992693

[9] K. Epp , R. Teipel , U. Reuner , M. Bornhäuser , C. Günther , and K. Trautmann‐Grill , “Successful Treatment of Scleromyxoedema With Daratumumab,” British Journal of Haematology 205, no. 1 (2024): 9–10, 10.1111/bjh.19366. 38471663

[10] L. Bettolini , S. Bighetti , V. Maione , I. Ghini , D. Segala , and P. Calzavara‐Pinton , “Successful Treatment of Scleromyxedema With Daratumumab, Lenalidomide and Dexamethasone in a Patient With Multiple Myeloma,” Australasian Journal of Dermatology 65, no. 4 (2024): e104–e107, 10.1111/ajd.14218. 38326991

[11] G. Bertuglia , S. Bringhen , B. Bruno , G. A. Impalà , and M. D'Agostino , “M‐Protein‐Related Necrobiotic Granuloma in a Multiple Myeloma Patient Treated With Daratumumab, Lenalidomide and Dexamethasone,” British Journal of Haematology 206, no. 1 (2025): 16–17, 10.1111/bjh.19887. 39543460 PMC11739761

[12] R. S. Petersen , L. M. Fijen , L. E. Franssen , J. M. I. Vos , and D. M. Cohn , “Daratumumab‐Based Treatment of Monoclonal Gammopathy–Associated Angioedema due to Acquired C1‐Inhibitor Deficiency,” Journal of Allergy and Clinical Immunology: Global 3, no. 4 (2024): 100322, 10.1016/j.jacig.2024.100322. 39282617 PMC11393585

